# Water Treatment from MB Using Zn-Ag MWCNT Synthesized by Double Arc Discharge

**DOI:** 10.3390/ma14237205

**Published:** 2021-11-26

**Authors:** Faizah S. Aljohani, Mohamed Elsafi, Nourhan I. Ghoneim, M. Toderaş, M. I. Sayyed, Hamidreza Mohafez, Mohammad A. Islam, Mayeen Uddin Khandaker, Mostafa El-Khatib

**Affiliations:** 1 Department of Chemistry, College of Science, Taibba University, Madinah P.O. Box 344, Saudi Arabia; m.sfm@hotmail.com; 2 Physics Department, Faculty of Science, Alexandria University, Alexandria 21511, Egypt; 3 Maritime Department, International Maritime College Oman (IMCO), Sohar 322, Oman; norhan@imco.edu.om; 4 Department of Physics, University of Oradea, 410 087 Oradea, Romania; monicatoderas@gmail.com; 5 Department of Physics, Faculty of Science, Isra University, Amman 11622, Jordan; dr.mabualssayed@gmail.com; 6 Department of Nuclear Medicine Research, Institute for Research and Medical Consultations (IRMC), Imam Abdulrahman Bin Faisal University (IAU), Dammam P.O. Box 1982, Saudi Arabia; 7 Department of Biomedical Engineering, Faculty of Engineering, Universiti Malaya, Kuala Lumpur 50603, Malaysia; 8 Department of Electrical Engineering, Faculty of Engineering, Universiti Malaya, Kuala Lumpur 50603, Malaysia; aminul.islam@um.edu.my; 9 Centre for Applied Physics and Radiation Technologies, School of Engineering and Technology, Sunway University, Bandar Sunway 47500, Malaysia; mayeenk@sunway.edu.my; 10 Basic Sciences Department, Faculty of Engineering, Pharos University in Alexandria, Alexandria 21526, Egypt

**Keywords:** double arc discharge, zinc-silver nanoparticles, carbon nanotubes, methylene blue, contaminated water, dye removal

## Abstract

A new type of nano-adsorbent zinc-silver nanoparticles ornamented by multi-walled carbon nanotubes (Zn-Ag MWCNT) was efficiently synthesized by double arc discharge using a newly designed rotating cylinder electrode. Zn-Ag MWCNT was characterized by different instrumental methods to get information about the sample shape, size, and crystallinity. Without irradiation, Zn-Ag MWCNT indicated significant potential for elimination against methylene blue (MB) which is dissolved in deionized water. When the adsorbent concentration was 0.1 g/L at normal 8 pH, the Zn-Ag MWCNTs were efficient in removing 97% of the MB from 40 mg/L that was dissolved in water for 10 min. The dye removal activity of (Zn-Ag) decorated MWCNTs was attributed to the influence of silver and zinc nanoparticles on the MWCNTs. Finally, this approach was both cost-effective and efficient.

## 1. Introduction

World populace increment caused inadequate access to consumable water, universally; more than 2.2 billion individuals utilize a drinking water sullied source [1]. Half of the world’s population will be suffering from a severe shortage of water resources in a few years [2]. Purification of water is one of the most significant applications, especially in nations with limited water resources. One of our country’s official goals is to reuse every drop of water, even multiple times. Many research laboratories suggested treating the toxic dye removal from water using different techniques such as photocatalytic degradation [3], Fenton’s oxidation [4], enzyme degradation (biological), and adsorption (physical) dye [5]. One of the popular applications is the adsorption process due to its simplicity. Trace of dyes in water, if it crosses the World Health Organization (WHO) thresholds, can trigger disease [1]. To solve this problem, scientists have used nanoparticles to remove dyes. Various types of adsorbents have been used in different settings to easily separate the dye from treated water. It is very important to study the applications of the prepared nanomaterials in solving some serious problems like water treatment. Arc discharge technique benefits from distinct advantages such as simple architectural design, utilizing non-toxic materials, and low cost adsorbents with high performance [6]. It is widely understood that silver nanoparticles (AgNPs) and these nano-families appear more prominent catalytic movement within the region of MB removal because of shape and size-dependent plasmon resonance [7]. It was discovered that the AgNPs in the products act as an e-transport link between the nano-adsorbent and the MB [8]. In latest years, carbonaceous compounds have received a lot of attention for adsorption applications, because of their exact stability, structural variety, density, and appropriateness for large-scale production, the adsorption of different types of dye may be a convenient alternative for graphene [9,10,11]. Carbon nanotubes (CNTs) decorated by transition metal such as snaps can increase the activity by using the charge mediator mechanism [3]. As carbon nanotubes have high adsorption efficiency for synthetic dyes, they are regarded among the most hopeful adsorbents for wastewater detoxification [12]. MWCNTs were discovered to be effective in removing MB from aqueous medium [13,14]. It has been stated that the adsorption capability of MWCNTs for MB removal is greater when compared to CNTs [15]. Previous work showed that there exists a most suitable particle size of approximately 33 nm for optimal photocatalytic action [16]. This most effective particle size is resulting from an increment interior of the charge carrier recombination rate, which resists the significant growth springing up from the high specific surface area particularly for a relatively small particle size [16]. The particle shape design of zinc oxide nanoparticles (ZnO Nps) and the initial concentration of MB are essential factors in dye removal [17]. To the most excellent of our information, however, there was neither report dealing with the effects of Zn-Ag MWCNT on wastewater treatment. Nanomaterials have different characterization than others in the form of clusters that may play a vital role in the review of the field of material sciences. The way of preparation can be chemically or physically [7,8,9]. Physical arc discharge method seems to be an alternative that is cheap, effective, and environmentally friendly. It may appear too simple and avoid chemical toxicity, and oxidizing nanometals [6]. Many research laboratories use two electrodes of arc discharge to produce metal nanoparticles or use two different metals to produce metal-metal nanocomposite [10]. A new design for preparation, a sample was created using double arc discharge successfully produces controlled size Zn-Ag MWCNT avoiding chemical toxicity. This novel study began with the creation of a Zn-Ag MWCNT nanocomposite using a rotational cylindrical cathode made of carbon arcing by two different anodes, one made of silver and the other of zinc, and then yield shape and size investigated using various characterization methods. Zn-Ag MWCNT nanocomposite activity on MB dissolved in deionized water under various conditions examined. At first, the process started with studying the effect of contact time to get an equilibrium time point, then get the best initial concentration of MB and suitable adsorbent dosage at specific pH.

## 2. Materials and Methods

### 2.1. Materials Procured

Deionized water as a solution was purchased from the Institute of Graduate Studies & research in Alexandria Egypt and MB with a purity above 99% was purchased from Alpha Chemika company, Andheri West, Mumbai, India. A zinc rod Purity is 99.5% was purchased from The Egyptian Company for Zinc, Cairo, Egypt and 100.00% pure cylindrical graphite (Ultra carbon P.O. Box 747, MICHIGAN 48706, Bay, MI, USA). An alternating current (AC) power supply was applied. Silver electrode with a purity above 99% was purchased commercially from the local sellers in Turkey.

### 2.2. Experimental Setup of Methodology

**Figure 1:**
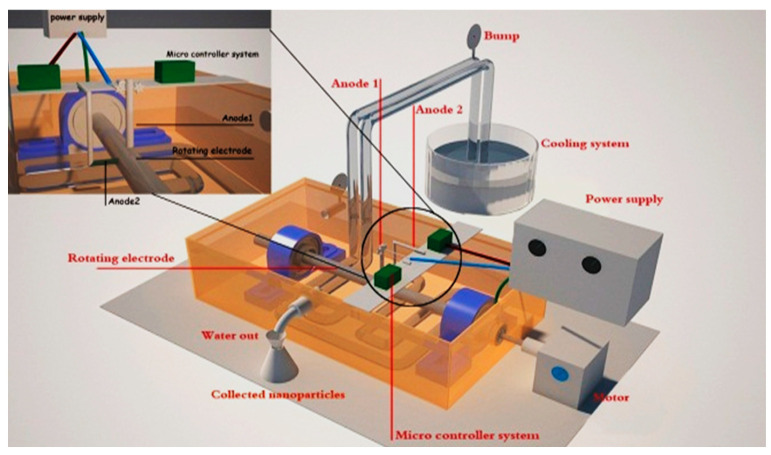
Electric arc discharge system.

In the present work double arc discharge unit was manufactured to be used to control one electrode, which is connected at 70 volts and current 15 A path through it first, while the second unit controls another electrode connected at 50 volts and 15 A current. Both electrodes act as anodes, which have dimensions 4 mm in diameter and 5 cm in length. This operation was carried out in a vessel containing 1 L of deionized water at 5.8 pH under the effect of atmospheric pressure. As demonstrated in Figure 1 where the whole system was modified from the previous work [10] by adding another feeding anode which is connected to a carbon cylindrical rotating cathode 16 mm rotated at 950 rpm in diameter and 5 cm in length.

The calibration process was carried out as follows: (1) 80 mg of MB as a decontaminated agent was added to 2 L of deionized water, (2) for homogeneity the decontaminated solution was ultrasonicated at room temperature for sufficient time, and (3) to determine MB concentration in water for any unknown sample, a calibration process was carried out by preparing well known concentrations (10, 20, 30, 35 and 40 mg/L) from the stock. UV-6800UV/VIS spectrophotometer (JENWAY-Germany) was used to study the absorbance versus the MB concentration. The sensitivity of the spectrophotometer was satisfactory.

### 2.3. Preparation of Zn-Ag MWCNT

The double arc discharge method was used to synthesize Zn-Ag MWCNT. Throughout synthesis of the nanocomposite, the arc discharge was generated between Ag and Zn electrodes. Both appear as anodes and pure carbon electrode appearing as a cathode as proven in Figure 1. High purity silver rod was used as the anode with a flat surface to keep uniform arcing during the experiment. Another zinc rod was drilled into a plexi container making an angle of 90° with the carbon electrode to produce the best yield [18]. Both silver and zinc electrodes were separated by a small distance gap carbon rotatory electrode; its motion was adjusted by a microcontroller system to maintain continuous three phase arc discharge [19]. The voltage used to preserve a steady release arc discharge between (silver, carbon) and (zinc, carbon) electrodes were 70 V and 50 V respectively. The double arc discharge in which first arc (sampling plasma) was used to evaporate and atomize the first metal sample, while the second arc is used to excite the resultant particles to combine with the second metal nanoparticles and carbon cathode. The yield prepared its characterizations were investigated to get clear information about size, shape, crystallinity, morphology, purity, and types of bonds attached together [10]. Zn-Ag MWCNT was done in only one step without the use of catalyst precursors to avoid the chemical toxicity of nano adsorbents used in the water dye removal.

### 2.4. Characterization

The yield prepared its characterizations were investigated to get clear information about size, shape, morphology (using JEOL JEM-2100 high resolution transmission electron microscope (HRTEM, at Alexandria University in Egypt, Model JEOL-JSM-6360LA) crystallinity (using X-ray diffraction analyzer, (JEOL Ltd., Tokyo, Japan), purity using (energy dispersive X-ray (EDX, JEOL Ltd., Tokyo, Japan) and types of bonds attached together (using Fourier-transform infrared at Egypt Japan University of Science and Technology, Alexandria, Egypt)

### 2.5. Adsorption Experiments of MB Dye

The procedure was followed in steps, and all experiments were done in triplicate, with the average values reported.

Factors Affecting the Adsorption Process

According to this calibration process, it was possible to study some factors affecting the process of water treatment as follow:

- Contact time: to find the best for removal of MB from contaminated water, 100 mg of the prepared Zn-Ag MWCNT as adsorbent dose was added to 100 mL MB solution (40 mg/L) in a dark bottle at fixed pH = 5.8 then the bottle was placed on an electrical shaker for a time extended to 35 min.
- Dye concentration: the previous experiment was repeated several times under the same conditions except for the concentration of MB in deionized water was varied from 10 mg/L to 40 mg/L. Each time the dye’s removal percent was determined by the UV spectrophotometer measurements.
- Nanomaterial’s Zn-Ag MWCNT dosage: it was worthy to study the effect of the adsorbant dosage on the water treatment from MB. Here the same experiment was repeated using dosage, ranging from 100 mg up to 300 mg.
- pH: this experiment was carried out at various pH using drops of NaOH or HCl.
- pH of the solution was measured using a pH meter after it had been balanced with NaOH or HCl solutions.
- Point of zero charge (pzc): the point of zero charge was determined according to Albis et al. [20]. In brief, 50 mL of 0.01 M NaCl was adjusted to pH from 2 to 12 at 1 pH unit interval by using 0.01 M NaOH and HCl. 0.1 g of the sorbents was added, and the mixture was stirred for 48 h. The pH of each batch was measured (pH meter: Hach Sension 1, model 51700-23, Shanghai, China). Initial and final pH values were recorded and plotted. Moreover, after each experiment, the nano adsorbent content was eliminated from water by centrifuge with a speed of 4000 rpm. All the tests were performed in duplicate. The reduced amounts of MB were calculated by the following equation [12]:

$$q=\frac{\mathrm{initial concentration}\left(c_{0}\right)-\mathrm{concentration at time}\;t\;\mathrm{of MB}\left(C_{t}\right)}{\mathrm{the volume of solution in liters}\left(v_{m}\right)}$$

In the desorption process, the MB dye adsorbed onto Zn Ag MWCNT was washed with ethanol, and water several times and transferred into a 100 mL beaker. The washing solution was also analyzed by UV to ensure the total desorption of MB. Then Zn Ag MWCNTwas reused several cycles to investigate the reusability.

## 3. Results and Discussions

### 3.1. Characterization of Synthesized Zn-Ag MWCNT

**Figure 2:**
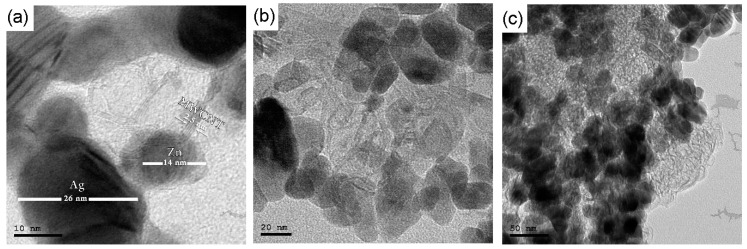
TEM image of Zn-Ag MWCNT (**a**) at 10 nm scale, (**b**) at 20 nm scale, and (**c**) at 50 nm scale.

TEM shows dark spherical particle shape of silver 24 ± 5 nm and hexagonal zinc at size with average diameter 33 ± 7 nm decorated by pale MWCNT. As shown in Figure 2 at the three different scales Ag and Zn surrounded MWCNTs have an outer diameter of about 3.5 ± 1.2 nm.

**Figure 3:**
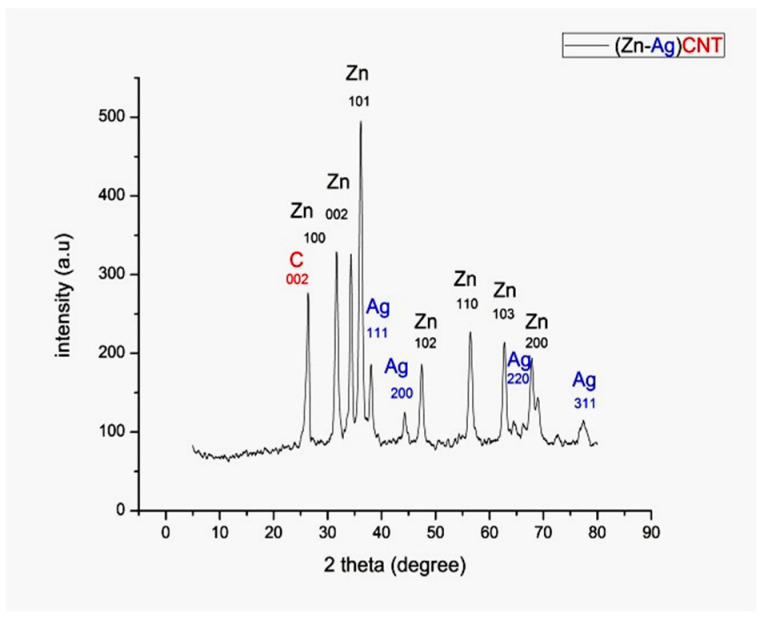
Shows the XRD patterns of Zn-Ag MWCNT.

The obtained XRD spectrum reveals main peaks as in Figure 3 which is in close agreement with the formation of Zn-Ag MWCNT. The XRD designs of all the tests show the high intensity of zinc peaks which is related to increases in zinc percentage in the sample prepared. The presence of hexagonal shape of ZnNPs confirmed by appearing of 7 diffraction peaks as maintained in JCPDS card file No. 36-1451 [21]. The presence of excessive intensity silver height (111) relative to the other remaining 3 peaks suggests the presence of a spherical form of silver nanoparticles with the agreement of 04-0783 standard card of the JCPDS [22]. The first absorbed peak at 26.4° indicates the presence of CNT from JCPDS No. 01-0646 [23].

**Figure 4:**
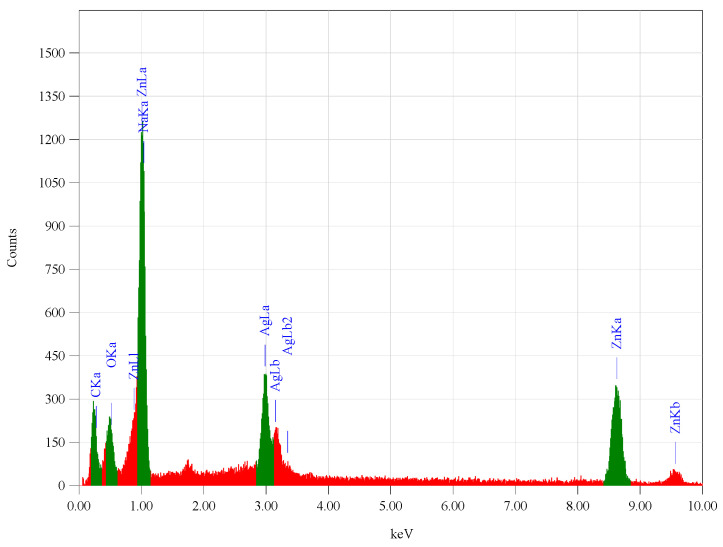
Shows EDX diagram.

The sample elemental purity was investigated using (EDX) to identify the elemental composition of the yield; the analysis is shown in Figure 4. It was observed that yields contained Zn, C, O, and Ag with percentages of 68.65%, 19.51%, 5.56%, and 6.28%, respectively.

**Figure 5:**
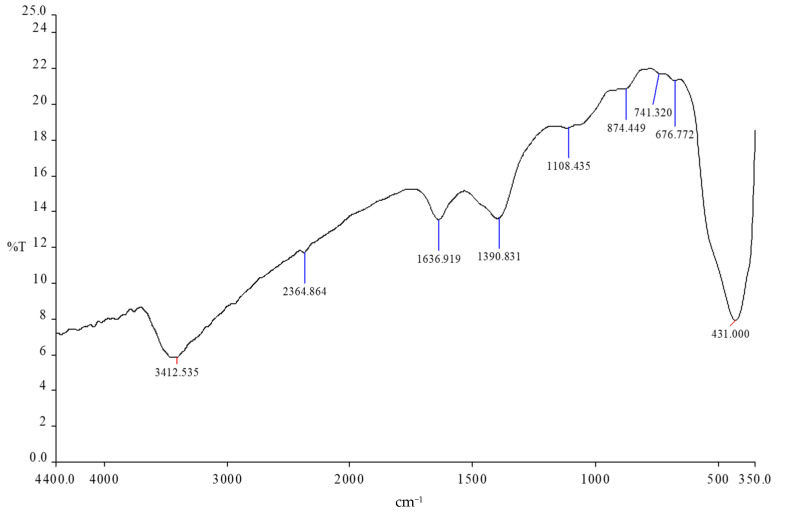
Shows the FTIR of Zn-Ag MWCNT.

To ensure the participating bond of Ag and Zn nano-metals with CNTs, FTIR was experimented as depicted in Figure 5. The peaks at 431 cm^−1^ [24] and 676 cm^−1^ [25] confirmed the structure of crystalline Zn-O and Ag-O NPs. The peaks around 3444 cm^−1^ and 2364 cm^−1^ [26] are due to -OH stretching vibration and O-H stretch from strongly hydrogen-bonded -COOH, respectively. The variation of other peaks (1638, 1460, and 1119 cm^−1^) [27,28] show the binding between ZnNPs and AgNPs and carbon skeleton tube through simple electrostatic attraction or formation of a coordination bond.

### 3.2. Water Decontamination Results

#### 3.2.1. Calibration of the Spectrophotometer Results

**Figure 6:**
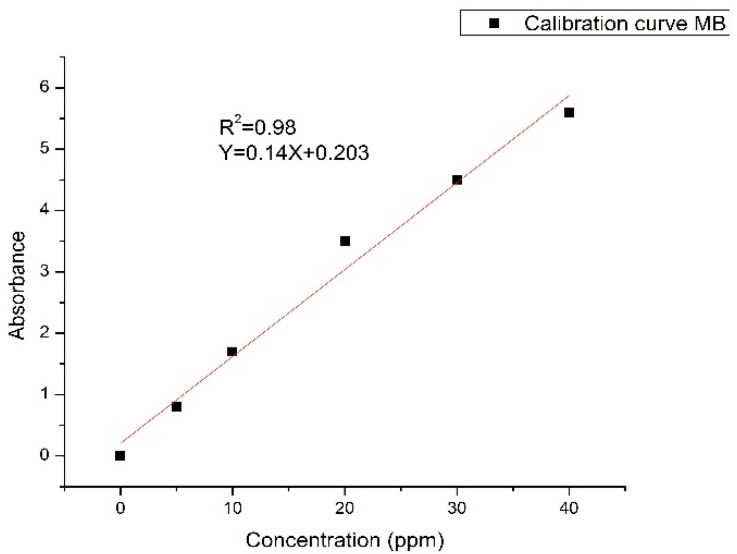
The variation of absorbance versus MB concentration.

To find the optimum condition for reducing MB, one studied the absorbance against the concentration of the MB. Standard samples of MB in deionized water with different concentrations were used to calibrate the spectrophotometer. The results revealed that the relation between absorbance and the concentration is linear as shown in Figure 6 given by:

absorbance(A)=molar absorptivity (Є) ×path length constants (l) ×sample concentration (C)

It was easy to get an empirical formula for the variation of the concentration with the absorbance.

(A) = 0.14 (C) (1)

#### 3.2.2. Impact of Contact Time

**Figure 7:**
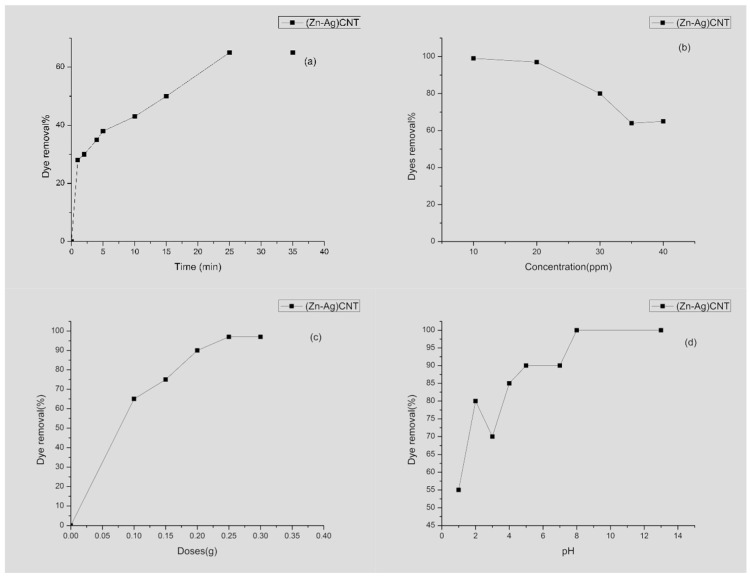
Effect of (**a**) contact time, (**b**) dye concentration, (**c**) adsorbent dose, and (**d**) pH on percent removal of MB.

To provide the color change, 100 mg of Zn-Ag MWCNT as a single adsorbent dose was added to 40 mg/L MB in 100 mL deionized water. The adsorbed quantity of MB steadily expanded with the contact time expanding until reaching the adsorption equilibrium time. It was clear from Figure 7a that the adsorption equilibrium time is ≈25 min and 65% MB dye is removed.

#### 3.2.3. Impact of Initial Dye Concentration

The introductory dye concentration has an impact on the decontamination process. On fixing the contact time at 25 min and the adsorbed dose of Zn-Ag MWCNT at 100 mg it was possible to study how much MB dye was removed from water by varying its concentration. Figure 7b revealed that at a low concentration, about 100% of the dye was removed, this removal percent decreases gradually as the concentration of dye increases to reach more than 65%.

#### 3.2.4. The Effect of Adsorbent Dosage

To enhance this performance and increase the dye removal, the study was expanded to use different doses of the absorbent agent Zn-Ag MWCNT. These doses were varied from 0.1 to 0.3 g/100 mL at MB beginning concentration 40 mg/L. Figure 7c; MB eliminating percentage steadily change with an adsorbent dosage until 0.3 g, after which the removal percentage almost reached a steady-state for nano-sorbents.

#### 3.2.5. Effect of pH

To examine the behavior of hydrogen power on MB, at the beginning 40 mg/L MB medium was disturbed with 0.1 g of Zn-Ag MWCNT at various pH values (1–13) using HCl and NaOH reagents at room temperature 27 °C and the data are depicted in Figure 7d; an increase in the% elimination of MB was observed for neutral and basic medium within contact time was 25 min. pH values much lower than point of zero charge (pHpzc) = 5.6, the surface of the MWCNTs will be more positive, and hence it will reduce its ability to interact with MB cation dye. As the pH increases, the negative charge on the Zn-Ag MWCNT surface provides electrostatic interactions with MB. According to these experiments, one can summarize as follows the best contact time was 25 min, the lowest dose 0.1 g for higher concentration of 50 ppm of MB gives dye removal exceeding 97% at neutral pH 8.

### 3.3. Kinetics Aspects

Laboratory adsorption results have been gotten for various times interims at incipient concentrations of 40 ppm MB dye was fitted to three kinetic models (pseudo-1st-order, pseudo-2nd order kinetic and Elovich model) in Table 1 for describing the function of the proposed adsorption process under optimum conditions [29]. Lagergren’s rate equation [30] was considered as one of the foremost broadly utilized rate equations to portray the adsorption of an adsorbate from the liquid stage.

The results show that the Pearson’s correlation “R” on Zn-Ag MWCNT adsorbents are low, and the non-reasonable discrepancy between the experimental and measured adsorption potential (*q_e_*) of Elovish and 1st order kinetic models demonstrated that this model fails to grasp the experimental evidence. The pseudo-2nd-order assumes that chemisorption is the rate-determining stage. The adsorption results have a high regression coefficient (0.98) with a pseudo-2nd-order kinetic model rate constant of 5.8 × 10^3^ (g/mg·min), indicating that adsorption on Zn-Ag MWCNT matches the pseudo-2nd-order kinetic model. Diffusion models aid in determining the exact adsorption process. The liquid-solid sorption process describes the mechanism of nano-metal CNT adsorbent by major stages [34]: (a) Nano-adsorbent host metal ions from the bulk fluid to its outer surface. (b) Intra-particle diffusion is known as either pore diffusion or solid surface diffusion. The metal ions are diffused inside adsorbent pores in the first mechanism, while the active sites on the adsorbent’s surface absorb the metal ions in the second. The only rate-limiting step is intra-particle diffusion. Adsorption kinetics may be dominated by both intra-particle diffusion and film diffusion at the same time. Table 1 summarized the findings, which showed that MB adsorption on Zn-Ag MWCNT applied the intra-particle diffusion model.

### 3.4. Isotherm Investigation

Adsorption isotherms are fundamental conditions for designing adsorption schemes. To interpret the equilibrium data in this analysis, the Langmuir, Freundlich, and Temkin isotherms were evaluated. The Langmuir isotherm is used to explain adsorption mechanisms and is based on the premise that uptake happens on a homogeneous surface through monolayer sorption with no interaction between adsorbed molecules. The value of separation factor $R_{L}$ indicates either the adsorption isotherm to be unfavorable ($R_{L}$> 1), favorable (0 < $R_{L}$> 1), linear ($R_{L}$= 1) or irreversible ($R_{L}$= 0). The $R_{L}$ values of Zn-Ag MWCNT adsorbent lies in the range of zero to unity indicating the favorable adsorption of MB dye on the adsorbent [35].


$$R_{L}=\frac{1}{1+k_{L}\times c_{o}}$$

The theoretical Freundlich study is premised on the hypothesis of the multilayer adsorbate formation on the adsorbent heterogeneous solid surface and concludes that the stronger binding sites are occupied first and that binding strength reduces with increasing degree of site occupation [36]. Values $R_{F}$ and $n_{F}$ are Freundlich constants that contribute to adsorption capacity and intensity of adsorption, respectively. The value of $n_{F}$ changes with the adsorbent’s heterogeneity, and for a desirable adsorption operation, the value of *n* should be less than 10 and greater than unity. As shown in Table 2 the value of n_f_ obtained was 2.3 for Zn-Ag MWCNT indicating a favorable adsorption process. Unlike the Langmuir and Freundlich isotherms, the Temkin isotherm model considers interactions between adsorbents and metal ions to be adsorbed and focuses on the premise that the free energy of sorption is a property of surface coverage [37].

The correlation coefficients, R^2^ values, were used to assess the applicability of the isotherm equation to explain the adsorption mechanism. The data were fitted by adsorption isotherm models in the following order: Langmuir > Temkin > Freundlich. The correlation coefficients, R^2^ values, were used to assess the applicability of the isotherm equation to explain the adsorption mechanism. The data were fitted by adsorption isotherm models in the following order: Langmuir > Temkin > Freundlich. The preceding order showed that two-parameter models matched the equilibrium data better. The Langmuir isotherm model suits the equilibrium results better, implying that the surface of Zn, Ag MWCNT for MB adsorption is heterogeneous. Furthermore, the Freundlich isotherm model has suggested multilayer adsorption of MB. The fact that the Langmuir isotherm closely matches the experimental results may be attributed to the homogeneous distribution of active sites. Due to the charge of MB, such a consequence is to be anticipated, so that the first charged adsorbent layer inhibits multiple layer adsorption.

Several approaches for incorporating co-catalysts have been suggested [38]. Carbon microspheres decorated with silver show interesting results to eliminate all the MB from an aqueous medium containing 30 mg/L MB within 60 s. When the microstructure was 0.12 g/L and exposed to visible light irradiation [39]. The photocatalytic activities of ZnO-coated MWNTs composite were analyzed to pure ZnO and pure MWNTs. The high adsorption of MB by MWNTs is due to their large specific surface area (185.3 m^2^ g^−1^) and cylindrical composition [40]. The photocatalytic activity of Ag-ZnO was stronger than that of reference titanium powders. The highest loss of MB occurred when the Ag loading was between 1% and 3%, and the reaction rate decreased as the Ag loading increased. It was found that 95% of MB was eliminated of the time in half-hour and 100% of the time in one hour 100% of the time in one hour [41]. The induced zinc and graphene in carbon nanotube nanocomposite with 3.9 wt% CNTs has a high adsorption capacity of 96 percent. The increased photocatalytic activity of the nanostructures composite is due to the increased optical emission and the decreased charge recombination caused by the addition of CNTs [42]. The adsorption capacity of Zn-Ag MWCNT was compared with others as tabulated in Table 3.

**Figure 8:**
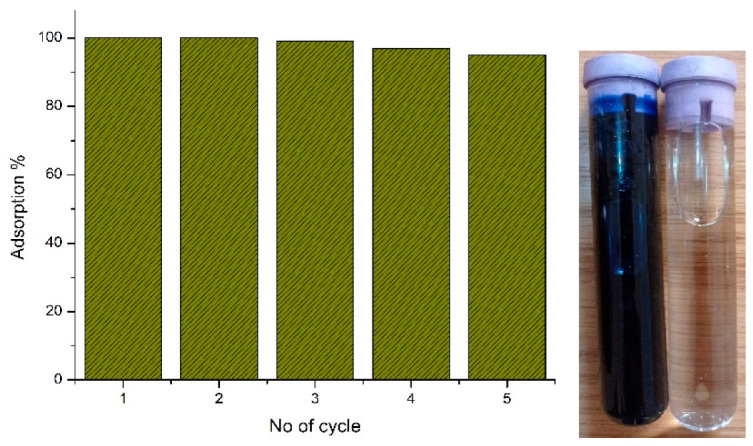
The reusability graph study and sample degradation after 5 cycle reuse.

According to the concept of sustainability, the reuse of nano adsorbent after use was examined by using the desorption process. After successful adsorption of Zn Ag MWCNT with MB occurred, the product of this combination was precipitated. The precipitated combined material was separated from the solution, washed with ethanol [52] to ensure desorption of MB from the nano adsorbent, and dried in air without structure damage for reuse in several cycles. The reuse study was carried out by repeating the adsorption–washing–desorption cycle five times under the same conditions to determine the removal efficiency in each cycle as shown in Figure 8.

The adsorbtion capacity of Zn, Ag MWCNT is compared with other materials prepared with the same method Ag-CNT, and ZnO NPs [10] showed that the Zn, Ag MWCNT is higher than ZnO NPs and lower than Ag-CNT. In the present work, the Ag% of Zn, Ag MWCNT reduced from 64.65% to 6.28% that present in Ag-CNT to not affect the toxicity of water. Additionally, Zn, Ag MWCNT can be regenerated and easily separated from aqueous solution without any weight loss. The Zn, Ag MWCNT prepared by the physical method has low toxicity compared with other materials prepared by chemical methods, is cheap, and is very effective in normal pH.

This suggests that Zn, Ag MWCNT are promising adsorbents for the removal of MB from aqueous systems.

## 4. Conclusions

This study offers a novel technique to prepare pure nano composites Zn-Ag MWCNT by a double arc discharge method using non-traditional rotation electrodes at a cost not exceeding one American dollar per each nano produced gram at the time of writing the search. It was fantastic to produce three different materials in the nano range companied together via using the physical method without any chemical toxicity which may affect portal water. The FTIR result confirmed the functioned MWCNTs obtained using various functional groups. The nano adsorbent under discussion was simple to make and demonstrated excellent MB dye adsorption capacity in polluted water at room temperature. It can be inferred that the best removal of MB dye from polluted water occurred at pH levels ranging from 8 to 12, an adsorbent dosage of 0.1 g/L, and a contact period of 25 min. Dye concentration parameter has a great influence while it is high on the elimination percentage of the solution. According to the kinetics and isotherm analysis, the experimental absorption results align well with pseudo second-order kinetics and Freundlich isotherm signals of chemisorption and multilayer adsorption, respectively.

## Figures and Tables

**Table 1:** The adsorption of MB onto Zn-Ag MWCNT adsorbent factors studies.

Isotherms	Linear Expression	Plot	Parameters	R^2^	Calculated Parameters	Ref.
1st-order kinetic	$\mathrm{Log}\;(q_{e}\;$ $-q_{t}$ $)\;=\mathrm{Log}q_{e}$ $-\frac{k_{1}}{2.303}t$	$\mathrm{Log}\;(q_{t}$$-q_{t}$) vs. *t*	*q_t_* = exp(intercept) $k_{1}\;$= −(slope × 2.303)	0.88	$k_{1}$= 0.138 min^−1^ *q_e_* = 21.71	[30]
2nd-order kinetic	$\frac{t}{q_{t}}=\frac{1}{k_{2}q_{e}^{2}}+\frac{t}{q_{e}}$	$\frac{t}{q_{t}}$ vs. *t*	*q_e_* = (slope)^−1^ *k*_2_ = (slope)^2^ × (intercept)^−1^	0.98	*k*_2_ = 5.8 × 10^3^ (g/mg·min) *q_e_* = 27.91	[31]
Elovich	$q_{t}=\frac{1}{\beta }$$\ln(\alpha \;\beta )+\frac{1}{\beta }$ ln (*t*)	*q_t_* vs. ln (*t*)	*β* = slope, *α* = (slope)^−1^ exp(intercept/slope)	0.92	*α* =1.747 (mg/g·min) *β* = 4.430 (g/mg)	[32]
Intraparticle diffusion	*q_t_* = *k_int_ t*^1/2^ + *C*	*q_t_* vs. *t*^1/2^	*k_int_* = slope	0.99	*k_int_* =3.373 *C* = 7.359	[33]
Film diffusion process	$\ln(1-\frac{q_{t}}{q_{e}})$= −R’*t*	$\ln(1-\frac{q_{t}}{q_{e}})$ vs. *t*	R’ = −(slope)	0.86	R’ = 0.156 min^−1^	[34]

**Table 2:** Isotherms and their linearized expressions.

Isotherms	Linear Expression	Plot	Parameters	R^2^	Calculated Parameters	Ref.
Langmuir	$\frac{1}{q_{e}}=\frac{1}{k_{L}*q_{m}}*\frac{1}{c_{e}}+\frac{1}{q_{m}}$	$\frac{1}{q_{e}}$ $\mathrm{vs}.\frac{1}{c_{e}}$	$q_{m}$= (intercept)^−1^ $K_{L}$= intercept/slope	0.995	$q_{m}$= 33.11 mg/g $K_{L}$= 0.250 L/mg	[35]
Freundlich	$\ln\;(q_{e}$ $)\;=\ln\;(K_{f})$ $+\frac{1}{n_{f}}$ $\ln\;(C_{e})$	$\ln\;(q_{e}$ $)\;\mathrm{vs}.\ln\;(C_{e})$	$K_{F}$= exp(intercept) *n_f_* = (slope)^−^^1^	0.910	$K_{F}$= 5.568 (mg/g)(L/mg)^1/*n*^ $n_{F}$= 2.358	[36]
Temkin	$q_{e}=q_{m}\ln k_{T}+q_{m}\ln C_{e}$	$q_{e}$ $\mathrm{vs}.\ln\;(C_{e})$	$q_{m}$= slope $K_{T}$= exp (intercept/slope)	0.959	$q_{m}$= 7.1031 mg/g $K_{T}$= 1.22 L/g	[37]

**Table 3:** Summary of the recently published articles for removal of MB dye from wastewater.

Adsorbent	Prepared Method	Adsorption Capacity (mg g^−1^)	Reference
Ag-CNT	Physical Arc discharge	45.87	[10]
ZnO NPs	Physical Arc discharge	25.12	[10]
MWCNTs	Chemical Method	95.30	[43]
Magnetic cellulose/GO composite	Chemical Method	70.03	[44]
Nano-Co_3_O_4_/SiO_2_	Chemical Method	53.87	[45]
Graphene oxide–Fe_3_O_4_ hybrid nano-composite	Chemical Method	167.20	[46]
Copper hydroxide nanowires decorated on activated carbon	Chemical Method	139.9	[47]
Carbon nanotubes	Chemical Method	46.20	[48]
Polyaniline nanotubes base	Chemical Method	9.21	[49]
Titanate nanotubes	Chemical Method	133.33	[50]
G–CNT hybrid	Chemical Method	81.97	[51]
Zn-Ag MWCNT	Physicsl Arc discharge	33.11	The present work

## Data Availability

The data presented in this study are available on request from the Corresponding authors.
